# Dual‐Function Nanoscale Coordination Polymer Nanoparticles for Targeted Diagnosis and Therapeutic Delivery in Atherosclerosis

**DOI:** 10.1002/smll.202401659

**Published:** 2024-08-26

**Authors:** Yuanzhe Lin, Jingjing Liu, Suet Yen Chong, Hui Jun Ting, Xichuan Tang, Liqiang Yang, Sitong Zhang, Xinyi Qi, Peng Pei, Zhigao Yi, Chenyuan Huang, Xiao Hou, Liang Gao, Federico Torta, Xiaogang Liu, Bin Liu, James Chen Yong Kah, Jiong‐Wei Wang

**Affiliations:** ^1^ Department of Surgery Yong Loo Lin School of Medicine National University of Singapore 1E Kent Ridge Rd Singapore 119228 Singapore; ^2^ Department of Biomedical Engineering National University of Singapore 4 Engineering Drive 3, Block E4, #04‐08 Singapore 117583 Singapore; ^3^ Nanomedicine Translational Research Program Yong Loo Lin School of Medicine National University of Singapore Singapore 117609 Singapore; ^4^ Institute of Translational Medicine Medical College Yangzhou University Yangzhou Jiangsu 225001 China; ^5^ Department of Chemical and Biomolecular Engineering National University of Singapore Singapore 117585 Singapore; ^6^ Cardiovascular Research Institute National University Heart Centre Singapore (NUHCS) 14 Medical Drive Singapore 117599 Singapore; ^7^ Department of Chemistry National University of Singapore 3 Science Drive 3 Singapore 117543 Singapore; ^8^ Department of Biochemistry Yong Loo Lin School of Medicine National University of Singapore Singapore 117596 Singapore; ^9^ Singapore Lipidomics Incubator (SLING) Life Sciences Institute National University of Singapore Singapore 117456 Singapore; ^10^ Department of Physiology Yong Loo Lin School of Medicine National University of Singapore 2 Medical Drive Singapore 117593 Singapore

**Keywords:** atherosclerosis, magnetic resonance imaging, nanoscale coordination polymers, pH‐responsive, theranostic

## Abstract

Atherosclerosis is the primary cause of cardiovascular events such as heart attacks and strokes. However, current medical practice lacks non‐invasive, reliable approaches for both imaging atherosclerotic plaques and delivering therapeutic agents directly therein. Here, a biocompatible and biodegradable pH‐responsive nanoscale coordination polymers (NCPs) based theranostic system is reported for managing atherosclerosis. NCPs are synthesized with a pH‐responsive benzoic‐imine (BI) linker and Gd^3+^. Simvastatin (ST), a statin not used for lowering blood cholesterol but known for its anti‐inflammatory and antioxidant effects in mice, is chosen as the model drug. By incorporating ST into the hydrophobic domain of a lipid bilayer shell on NCPs surfaces, ST/NCP‐PEG nanoparticles are created that are designed for dual purposes: they diagnose and treat atherosclerosis. When administered intravenously, they target atherosclerotic plaques, breaking down in the mild acidic microenvironment of the plaque to release ST, which reduces inflammation and oxidative stress, and Gd‐complexes for MR imaging of the plaques. ST/NCP‐PEG nanoparticles show efficacy in slowing the progression of atherosclerosis in live models and allow for simultaneous in vivo monitoring without observed toxicity in major organs. This positions ST/NCP‐PEG nanoparticles as a promising strategy for the spontaneous diagnosis and treatment of atherosclerosis.

## Graphical abstract

A dual‐function nanoscale coordination polymers‐based theranostic system, ST/NCP‐PEG, is synthesized for managing atherosclerosis. This system can efficiently co‐deliver gadolinium and simvastatin (ST) to the plaque and release both cargos therein in response to the acidic microenvironment, enabling spontaneous magnetic resonance imaging and efficacious treatment of atherosclerosis. Moreover, this system is biocompatible, biodegradable, and does not exert obvious toxicity.

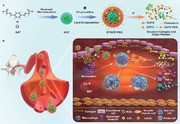

## 1 Introduction

Atherosclerosis is a chronic inflammatory disease characterized by the accumulation of cholesterol on the walls of blood vessels. Rupture of atherosclerotic plaques is the main cause of acute cardiovascular events, which accounted for 32% of global deaths in 2019.^[^ ^1^ ^]^ Therefore, a sensitive and reliable diagnostic strategy is crucial to improve atherosclerosis management. Intravascular ultrasound (IVUS) and computed tomography angiography (CTA) are clinical gold standards in visualizing atherosclerotic plaques. However, invasive IVUS is prone to cause injury and complications.^[^ ^2^ ^]^ CTA can only evaluate arteries larger than 1 mm in diameter since the iodinated contrast agent flows rapidly within arteries,^[^ ^3^ ^]^ leaving the microvasculatures, which are ubiquitous in the plaques, inaccessible. Furthermore, the microvasculature is prone to rupture due to lack of mural cells and poorly formed endothelial cell junctions, increasing the risk of acute cardiovascular events. Statins, HMG‐CoA reductase inhibitors, are widely prescribed to lower cholesterol levels in order to prevent or treat cardiovascular disease. Apart from lowering cholesterol levels, statins such as simvastatin (ST) have been suggested to exert anti‐inflammatory and antioxidative activities that partly contribute to their anti‐atherosclerosis effects.^[^ ^4^, ^5^, ^6^ ^]^ However, this therapy is not always effective in all population of patients due to its hydrophobic properties, non‐specific distribution and rapid excretion following oral intake. Therefore, there has been a trend toward intensive statin dosage,^[^ ^7^, ^8^, ^9^ ^]^ leading to dose‐dependent adverse events such as myopathy, hepatoxicity, and type 2 diabetes.^[^ ^10^, ^11^ ^]^

The advent of nanotechnology has revolutionized the treatment landscape for many diseases. Nanoparticles can extravasate from the bloodstream and readily accumulate in atherosclerotic plaques through the permeable endothelium of the inflamed arteries and thereby increasing plaque concentration of contrast agents for enhancing diagnosis signal.^[^ ^12^, ^13^ ^]^ Moreover, nanoparticle‐based drug delivery systems have been demonstrated to be an effective strategy to improve therapeutic outcomes in atherosclerosis by improving the bioavailability and local concentrations of therapeutic agents.^[^ ^14^, ^15^ ^]^ Therefore, many types of nanomaterials have been explored to develop nanomedicines for improvement in the diagnosis and treatment of atherosclerosis in recent years.^[^ ^16^, ^17^ ^]^ Nanoscale coordination polymers (NCPs) are an emerging hybrid nanomaterial which are constructed by bridging metal ions and organic linkers. Due to their intrinsic biodegradability and structural tunability, NCPs can be synthesized with different sizes, compositions, and chemical properties for various biomedical applications.^[^ ^18^ ^]^ Furthermore, the porous structure of NCPs allows for high payload of various types of therapeutic agents.^[^ ^19^, ^20^, ^21^, ^22^ ^]^ However, biocompatible and biodegradable NCPs based nanomedicine for managing atherosclerosis has not been reported.

**Scheme 1:**
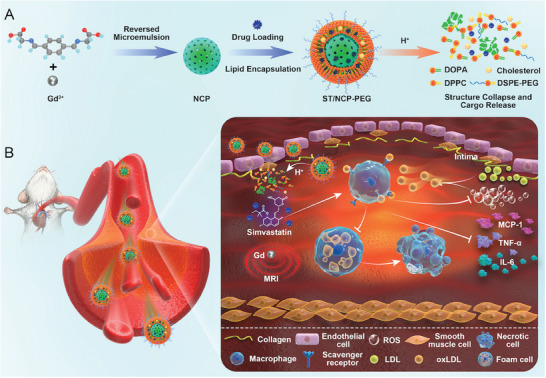
Schematic illustration of A) fabrication procedure of ST/NCP‐PEG and pH‐responsive structural collapse under acidic microenvironment. B) the mechanism of pH‐responsive and MRI‐functional ST/NCP‐PEG nanomedicine enabling spontaneous diagnosis and treatment of atherosclerosis.

Here, we aimed to develop a NCPs‐based pH‐responsive dual‐function nanomedicine for spontaneous imaging and treatment of atherosclerosis (**Scheme** **1**). We first synthesized NCPs with a pH‐responsive benzoic‐imine linker (BI‐linker) and Gadolinium (Gd^3+^). NCPs were then modified with PEGylated lipid bilayer to achieve better colloidal stability and longer blood circulation time. ST was loaded into the porous structure of NCPs and the hydrophobic domain of lipid bilayer shell on the surface of NCPs to formulate ST/NCP‐PEG nanoparticles. These nanoparticles exerted anti‐inflammatory and antioxidant activities in vitro attributed to ST. By design, these nanoparticles remained intact under physiological conditions (neutral pH) but decomposed rapidly in response to the mild acidic microenvironment in lipid‐rich atherosclerotic plaques,^[^ ^23^, ^24^ ^]^ resulting in a prompt release of ST and Gd complexes. Upon intravenous administration, ST/NCP‐PEG nanoparticles efficiently accumulated in the plaques and accordingly enriched both ST and Gd complexes therein, which enabled spontaneous magnetic resonance imaging (MRI) of plaques and efficacious treatment of atherosclerosis. In addition, no toxicity in major organs were detected.

## 2 Results and Discussion

### 2.1 Preparation and Characterization of ST/NCP‐PEG Nanoparticles

**Figure 1:**
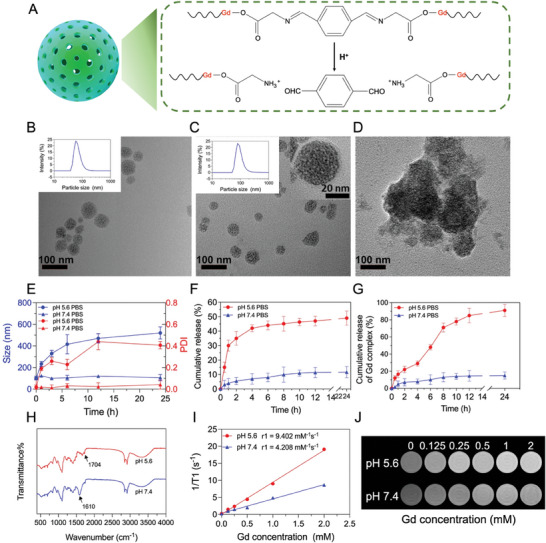
Characterization of ST/NCP‐PEG nanoparticles. A) The schematic illustration for the pH‐responsive dissociation of NCP nanoparticles. TEM images of B) NCP (the inserted graph was hydrodynamic diameter (D_h_) of NCP), C) NCP‐PEG nanoparticles (the inserted graph was D_h_ of NCP‐PEG) and D) NCP‐PEG nanoparticles decomposed in pH 5.6 PBS after 4 h. E) Size and PDI of NCP‐PEG in pH 5.6, pH 7.4 PBS at different time points. The cumulative release of ST F) and Gd complex G) from ST/NCP‐PEG nanoparticles at pH 5.6 and pH 7.4. H) FT‐IR spectrum of NCP‐PEG nanoparticles at pH 5.6 and pH 7.4 for 4 h. r1 relaxivities I) and T_1_‐weighted MR images J) of NCP‐PEG nanoparticles at pH 5.6 and pH 7.4. Data is presented as mean ± SD (E‐G, *n* = 3; each experiment was repeated 3 times independently, *n* represents the number of repetitions per group).

The synthetic procedure of pH‐responsive NCP‐PEG nanoparticles and drug loading process were illustrated in Scheme 1A and **Figure** **1**A. As assessed by dynamic light scattering (DLS) and transmission electron microscopy (TEM), NCP nanoparticles exhibited uniform size and spherical shape with an average hydrodynamic diameter of ≈40 nm (Figure 1B). In addition, the composition of NCP nanoparticles was determined with thermo‐gravimetric analysis (Figure S1A, Supporting Information) and inductively coupled plasma mass spectrometry (ICP‐MS). The mass ratios of BI‐linker and Gd were 53.2 and 39.2 wt.%, respectively.

In order to improve colloidal stability, dispersibility, and biocompatibility, lipid bilayers were coated on NCP nanoparticles per previous described with minor modifications.^[^ ^25^ ^]^ Basically, a monolayer of 1, 2‐dioleoyl‐sn‐glycero‐3‐phosphate (sodium salt) (DOPA) was first coated on NCP nanoparticles through the coordination interaction between phosphate groups in DOPA and Gd ions in NCPs. Then the obtained NCP‐DOPA was dispersed in chloroform and modified with additional lipids, 1, 2‐dihexadecanoyl‐sn‐glycero‐3‐phosphocholine (DPPC), cholesterol and 1,2‐distearoyl‐sn‐glycero‐3‐phosphoethanol‐amine‐N‐(methoxy(polyethyleneglycol)−5000) (DSPE‐mPEG_5k_) via hydrophobic interactions to obtain NCP‐PEG nanoparticles. As shown in Figure 1C, NCP‐PEG nanoparticles exhibited a clear and relatively low‐contrast lipid shell on top of nanoparticle surface. There was no notable morphology and size change after lipid bilayer modification according to the TEM images (Figure 1B,C). The hydrodynamic diameter (D_h_) of NCP‐PEG nanoparticles was slightly larger than that of NCPs (79 ± 2 nm vs 59 ± 2 nm; Figure S1B, Supporting Information), indicating the coating of PEG.

ST is known to attenuate atherosclerosis partly because of its potent anti‐inflammatory and antioxidant properties.^[^ ^4^, ^6^ ^]^ However, the hydrophobic property of ST greatly limits its clinical potential. The porous structure of NCPs^[^ ^26^, ^27^ ^]^ and the hydrophobic domain within the lipid bilayer coating provide desirable space for ST loading, preventing precipitation of ST in the aqueous environment. As shown in Figure S1C (Supporting Information), the drug loading (DL%) capacity of ST/NCP‐PEG nanoparticles was 44.8 ± 1.9% with a molar ratio of DPPC, cholesterol, ST, and DSPE‐mPEG_5k_ at 4:4:3:2.

Atherosclerotic plaques are hypoxic.^[^ ^28^, ^29^ ^]^ Although the pH values in human atherosclerotic plaques were reported highly heterogenous, acidic pH values were detected in highly inflammatory and lipid‐rich plaques.^[^ ^23^, ^24^, ^30^, ^31^ ^]^ Activated macrophages within the plaques have a high energy demand and thus use glycolysis for ATP synthesis under hypoxia conditions, which increases the secretion of lactate and protons,^[^ ^23^, ^32^ ^]^ leading to an acidic microenvironment. To enhance drug delivery efficiency in the highly inflammatory and lipid‐rich plaques, we designed NCP‐PEG nanoparticles with a pH‐responsive linker, benzoic‐imine (BI) linker. The BI linker within NCPs is stable at pH 7.4 but easily hydrolyzed under acidic environment. Therefore, we first investigated the structural collapse behavior of NCP‐PEG nanoparticles by measuring hydrodynamic diameter (D_h_) and polydispersity index (PDI) under different conditions. We observed no significant D_h_ change of NCP‐PEG nanoparticles at pH 7.4 (Figure 1E), indicating the desirable stability of NCP‐PEG nanoparticles under neutral environment. At pH 5.6, however, the D_h_ and PDI of NCP‐PEG nanoparticles drastically increased over the 24 h incubation period, indicating the progressive structural collapse. Similar structural collapse of NCP‐PEG nanoparticles was observed under TEM after incubation in acidic PBS (pH 5.6) for 4 h (Figure 1D).

The pH‐responsiveness of NCP‐PEG nanoparticles described above is desirable to achieve controlled drug release and MRI signal enhancement within the mild acidic plaque microenvironment. To confirm the desired properties of NCP‐PEG nanoparticles, we first evaluated the in vitro ST release performance from ST/NCP‐PEG nanoparticles under different conditions (Figure 1F). Under physiological conditions with pH 7.4, the release of ST was constant at a slow rate, cumulatively reaching 11.7% during the 24 h incubation period. In contrast, at pH 5.6, a burst release of ST occurred, cumulatively reaching 35%, within 2 h, ≈7 times faster than the release under pH 7.4. As a result, the cumulative release reached as high as 49% after 24 h, which is 4 times higher than that at pH 7.4. This drug release feature of ST/NCP‐PEG nanoparticles is designed to facilitate efficient delivery of ST to the atherosclerotic plaque with minimal drug loss while circulating in the blood. As expected, the Gd complex release profile was similar to that of ST (Figure 1G), further demonstrating the NCPs structural collapse preferentially under acidic conditions. The slightly faster release of ST than Gd^3+^ in the first 4 h is likely because ST is loaded in the outer lipid bilayer and the porous structure of NCPs, whereas Gd^3+^ is part of the NCP polymer structure (Scheme 1A), which takes longer time to break down. Nonetheless, to elucidate the pH‐responsiveness mechanism of NCPs, Fourier‐transform infrared (FTIR) was conducted (Figure 1H). A decrease in pH from 7.4 to 5.6 led to a notable reduction in the stretching vibration of the imine bond at 1610 cm^−1^, whereas a sharp peak at 1704 cm^−1^ attributed to aldehyde bond emerged, indicating the cleavage of BI‐linker in NCPs. The released Gd‐complex provided increased accessibilities to adjacent water molecules, resulting in a heightened *T_1_*‐weighted MRI signal. As depicted in Figure 1I,J, the longitudinal relaxivity (*r_1_* value) at pH 7.4 was 4.208 mm ^−1 ^s^−1^. Conversely, the *r_1_* value surged to 9.402 mm ^−1 ^s^−1^ at pH 5.6, indicating the target‐specific MRI “on‐off” potential of NCP‐PEG nanoparticles. Taken together, despite various nanoparticle systems have been developed for diagnosis and/or treatment of atherosclerosis,^[^ ^16^, ^17^ ^]^ we here developed, to our best knowledge, for the first time a NCPs based and acidic plaque microenvironment‐responsive theranostic nanomedicine for atherosclerosis therapy.

### 2.2 In Vitro Antioxidant and Anti‐inflammatory Effects in Macrophages

**Figure 2:**
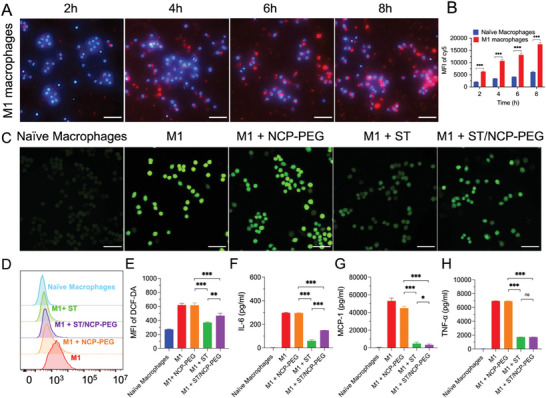
Cellular uptake and biological activities of ST/NCP‐PEG nanoparticles in macrophages. A) Microscopic images showing time‐dependent cellular uptake of Cy5‐labeled NCP‐PEG nanoparticles by M1 macrophages. B) FACS quantification of MFI in RAW264.7 macrophages to assess cellular uptake of ST/NCP‐PEG nanoparticles at different incubation timepoints. C) Microscopic fluorescent images showing intracellular ROS detection by DCF‐DA assay. D,E) Quantification of intracellular ROS levels by cell mean fluorescence intensity (MFI) determined by FACS of cells after microscopic analysis in (C). Typical inflammatory cytokines IL‐6 F), MCP‐1 G), TNF‐α H) secreted by RAW264.7 cells. Equal concentration of ST (24 µm) was used either in free form or nanoformulations. Scale bars = 50 µm. Data is presented as mean ± SD (A‐E, *n* = 3; F‐H, *n* = 5; *n* represents the number of independent experiments per group). MFI, mean fluorescence intensity. ns, not significant; *p < 0.05, **p < 0.01, ***p < 0.001.

Macrophages play a critical role in atherosclerosis development^[^ ^33^ ^]^ and account for a large proportion of the immune cells within the plaque.^[^ ^34^ ^]^ Therefore, cellular uptake of Cy5‐labelled NCP‐PEG nanoparticles by macrophages was examined. As atherosclerotic plaques provide an inflammatory microenvironment with different pro‐inflammatory stimuli and thus contain abundant M1 macrophages,^[^ ^35^, ^36^ ^]^ we also evaluated whether M1 macrophages have enhanced cellular uptake of NCP‐PEG nanoparticles. Naïve macrophages not subject to stimulation were referred to as M0 macrophages, whereas those stimulated with lipopolysaccharide (LPS) and interferon‐γ (IFN‐γ) were referred to as M1 macrophages. As shown by inverted fluorescence microscopy, cellular uptake of Cy5‐labelled NCP‐PEG nanoparticles by both native and M1 macrophages increased in a time‐dependent manner (**Figure** **2**A; Figure S2A, Supporting Information). When quantified by FACS analysis, M1 macrophages showed a markedly higher cellular uptake of nanoparticles compared to naïve (or M0) macrophages (Figure 2B; Figure S2B,C, Supporting Information). These results are in agreement with the notion that M1 macrophages possess enhanced mitochondrial ATP synthesis^[^ ^37^ ^]^ and receptor Fc gamma expression,^[^ ^38^ ^]^ leading to incremental phagocytosis. Taken together, these results suggest that NCP‐PEG nanoparticles could target plaque lesional macrophages.

Reactive oxygen species (ROS) production at a relatively low level is important in keeping homeostasis of cellular microenvironment, however, excessive ROS may overwhelm the antioxidant system and activate proinflammatory signaling and therefore aggravate atherosclerosis progression.^[^ ^39^ ^]^ Since the therapeutic efficacy of ST in atherosclerosis is partly attributed to its anti‐inflammatory and anti‐oxidant bioactivities,^[^ ^40^, ^41^ ^]^ we hereby determined whether ST‐loaded formulations retain the antioxidant activity of ST at cellular level. After 4 h stimulation, M1 cells produced high levels of ROS, as indicated by DCF‐DA assay where green fluorescence is emitted upon reaction with ROS (Figure 2C). Treatment with NCP‐PEG nanoparticles did not change ROS levels in M1 cells, indicating that NCP‐PEG nanoparticles alone have no impact on ROS production in macrophages. In contrast, fluorescence intensity was significantly lowered in cells treated with either ST/NCP‐PEG or free ST. These microscopic findings were confirmed by quantitative analysis with FACS (Figure 2D,E). These results indicate that ST encapsulated in NCP‐PEG nanoparticles retains its antioxidant activity although the exact mechanisms remain unclear.^[^ ^40^ ^]^ It is worth noting that free ST showed better antioxidant efficacy than ST/NCP‐PEG nanoparticles. This is likely due to better access of cells to the lipophilic small molecule drug ST since surface PEGylation reduces cellular uptake of nanoparticles by macrophages^[^ ^42^, ^43^ ^]^ and ST/NCP‐PEG nanoparticles do not release ST in nonacidic environment such as the culture medium or cytosol (Figure 1F). Nonetheless, as ROS can activate multiple signaling pathways that aggravate atherosclerotic inflammation,^[^ ^44^ ^]^ antioxidant activity of ST/NCP‐PEG nanoparticles, in principle, would attenuate inflammation and therefore atherosclerosis.

Moreover, we investigated whether ST treatment could alleviate inflammatory responses in macrophages. As expected, M1 macrophages expressed higher interleukin‐6 (IL‐6), Monocyte Chemoattractant Protein‐1 (MCP‐1), and tumor necrosis factor (TNF‐α) than naïve macrophages (Figure 2F–H). Upon treatment of M1 macrophages with free ST or ST/NCP‐PEG (at an identical ST dose of 24 µm) for 24 h, the expression of those pro‐inflammatory cytokines was inhibited to various extents. Interestingly, ST/NCP‐PEG exerted a stronger inhibitory effect on MCP‐1 expression than free ST (Figure 2G). Given that MCP‐1 released from endothelial cells and smooth muscle cells aggravates atherosclerosis by recruiting monocytes to the subendothelial layer,^[^ ^45^, ^46^ ^]^ reduction of MCP‐1 expression by ST/NCP‐PEG may result in alleviation of atherosclerosis progression.

### 2.3 Accumulation of NCP‐PEG Nanoparticles Within Plaques Enables MRI‐Guided Diagnosis

**Figure 3:**
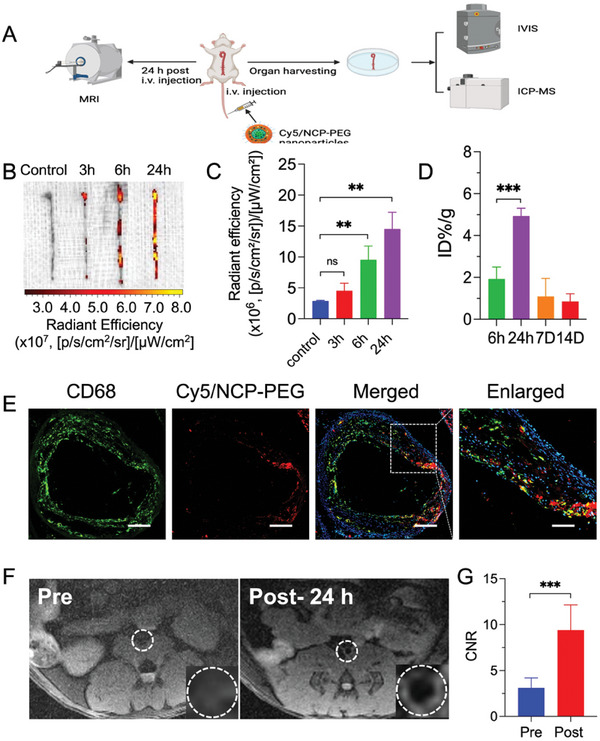
In vivo and ex vivo imaging of atherosclerotic plaques in ApoE^−/−^ mice. A) Schematic illustration of fluorescence and MRI imaging. B) Ex vivo fluorescent imaging of the aortas and C) quantitative data analysis of Cy5‐labeled fluorescent signals in the aorta. D) Distribution of Gd content in aorta at 6, 24, 7 days (7D), and 14 days (14D) post i.v. injection of NCP‐PEG nanoparticles. E) Immunofluorescence staining of macrophages (CD68 in green) in the aortic root. Cy5‐labeled NCP‐PEG nanoparticles were indicated in red. Co‐localization of macrophages and nanoparticles were indicated in orange. F) The *T_1_*‐weighted MR images in ApoE^−/−^ mice (the region of interest is outlined in the insert) and G) contrast to noise ratio (CNR) of aortic wall to lumen before (Pre) or 24 h after (Post) administration of NCP‐PEG nanoparticles. Data is presented as mean ±SD (B‐C, *n* = 4; D‐G, *n* = 5; *n* represents the number of mice per group). ns, not significant; **p < 0.01, ***p < 0.001.

While ST is widely prescribed, it has slow dissolution rate due to its hydrophobic nature. In addition, free drug molecules are usually non‐specifically distributed and rapid cleared upon intravenous administration, leading to a poor bioavailability and undesirable therapeutic outcomes.^[^ ^47^ ^]^ On the other hand, a very high dosage needed to achieve desirable therapeutic efficacy may cause severe adverse events.^[^ ^48^, ^49^ ^]^ Introduction of nanoparticles could improve the dissolution rate of hydrophobic drugs by increasing specific surface area and solubility.^[^ ^50^, ^51^, ^52^ ^]^ The inflamed endothelium in the plaque region is highly permeable,^[^ ^53^ ^]^ thus nanoparticles could accumulate within the plaques via the enhanced permeability and retention (“EPR”) effect,^[^ ^34^ ^]^ achieving targeted drug delivery and controlled release kinetics. In our current study, to achieve targeted delivery of ST and imaging of atherosclerotic plaques, we modified the surface of NCP nanoparticles by PEGylation which enables their long circulation in the bloodstream to fully employ EPR effects for plaque targeting.^[^ ^54^ ^]^ As expected, at 6 h post i.v. injection of Cy5‐labeled NCP‐PEG nanoparticles, the isolated whole aorta from plaque‐bearing ApoE^−/−^ mice exhibited obvious fluorescence signal in the aortic arch and abdominal aorta regions, where were prone to form plaques (**Figure** **3**B,C). At 24 h post‐injection, higher fluorescent signal was observed in the entire aorta, indicating accumulation of NCP‐PEG nanoparticles in the atherosclerotic lesions.

To confirm if Gd^3+^ ions were indeed delivered to the plaque lesions, Gd^3+^ content in excised aorta was quantitatively analyzed at different time points post i.v. injection of NCP‐PEG nanoparticles (Figure 3D). As assessed by ICP‐MS, accumulation of Gd element in the aorta reached the peak at 24 h post‐administration, which was aligned with the IVIS imaging (Figure 3B,C). Gd could barely be detectable in the major organs, urine, and blood after 14 days post‐administration, indicating that NCP‐PEG nanoparticles are degraded and excreted from the body. This feature is critical as long‐term retention of nanoparticles pose potential adverse events.

Since PEGylated nanoparticles are mostly taken up by macrophages in atherosclerotic plaques and the injured heart tissue,^[^ ^55^ ^]^ we also analyzed distribution of NCP‐PEG nanoparticles in the plaque at the cellular level. Similarly, Cy5‐labelled NCP‐PEG nanoparticles accumulated in the plaque were mostly co‐localized with macrophages (Figure 3E). The enhanced cellular uptake of NCP‐PEG nanoparticles by plaque lesional macrophages may be attributable to their long interaction and the high phagocytic activity of inflamed macrophages as indicated in Figure 2A,B. In light of the efficient delivery of Gd^3+^ to the plaque as evidenced above (Figure 3B–E), we then assessed the imaging performance of NCP‐PEG nanoparticles in vivo by carrying out MRI on 16 weeks old ApoE^−/−^ mice that were fed on HFD for 12 weeks. As depicted in Figure 3F, there was a remarkable signal enhancement of arterial vessel wall, indicating the presence of atherosclerotic plaques. Imaging of the atherosclerotic plaque was confirmed by notable increase in the contrast to noise ratio (CNR) which determines the contrast of vessel wall relative to the lumen (Figure 3G). These results demonstrate that NCP‐PEG nanoparticles accumulate specifically in the plaque and therefore enable detection of atherosclerotic plaques in the blood vessel by MRI.

### 2.4 ST/NCP‐PEG Nanoparticles Enable Spontaneous Treatment and Monitoring of Atherosclerosis in Mice

**Figure 4:**
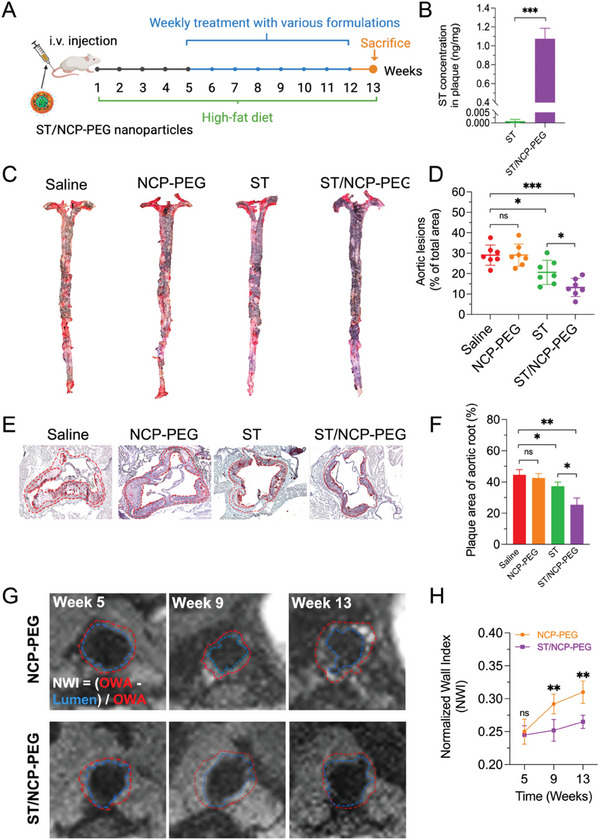
Theranostic effects of ST/NCP‐PEG nanoparticles on atherosclerosis in ApoE^−/‐^ mice. A) Schematic illustration of the experimental design. B) LC‐MS quantification of ST in the aorta plaque 24 h post‐injection. ST concentration was normalized to ng per mg of aortic tissue. C) Representative photographs of en face ORO‐stained aortas from mice receiving different treatments. D) Quantitative analysis of the lesion area in aortas. E) Images of aortic root sections stained with ORO and H & E. F) Quantification of plaque area relative to arterial wall area. G) The *T_1_*‐weighted MR images of abdominal aorta and H) vessel wall thickness expressed as the normalized wall index (NWI) at different times points. Data is presented as mean ± SD (B, *n* = 3; C‐D, *n* = 7; E‐H, *n* = 3; *n* represents the number of mice per group). ns, not significant; *p < 0.05, **p < 0.01, ***p < 0.001.

Since ST/NCP‐PEG nanoparticles were able to deliver MRI contrast agent to the plaque, we next assessed if this nanoformulation could improve in vivo therapeutic efficacy of ST as illustrated in **Figure** **4**A. To validate the drug delivery efficiency to the plaque by ST/NCP‐PEG nanoparticles, we first determined ST concentrations in the aortic tissue with LC‐MS at 24 h post i.v. administration when accumulation of nanoparticles peaked in the plaque (Figure 3B–D). As shown in Figure 4B, ST/NCP‐PEG nanoparticles increased plaque tissue concentrations of ST by more than 1000 times compared with i.v. administration of free ST. These results demonstrate that NCP‐PEG nanoparticles can deliver both the contrast agent and therapeutic to the plaque with high efficiency. To fully assess therapeutic efficacy of ST/NCP‐PEG nanoparticles in atherosclerosis, after receiving atherogenic high‐fat diet (HFD) for 4 weeks, ApoE^−/−^ mice were randomly allocated into 3 groups receiving weekly treatment of NCP‐PEG, free ST, and ST/NCP‐PEG, while fed on HFD for additional 8 weeks. The whole aortas were harvested after 8 weeks of treatment and stained with Oil red O (ORO) for histology analysis. As expected, mice received the vehicle control NCP‐PEG developed significant atherosclerosis plaques as indicated by ORO staining (Figure 4C). In contrast, both free ST and ST/NCP‐PEG treatment, at an identical ST dose of 2 mg kg^−1^, reduced atherosclerotic lesion size. In particular, the average lesion area of ST/NCP‐PEG group was 36% and 54.6% smaller than that of free ST and NCP‐PEG groups, respectively (Figure 4D). Since atherosclerosis development starts from the aortic sinus and plaque lesion volume at the aortic roots is used as an additional parameter to assess atherosclerosis progression.^[^ ^56^ ^]^ As demonstrated in Figure 4E,F, in concordance with the whole aorta data, plaque lesion volume at the aortic roots was markedly reduced by ST/NCP‐PEG treatment.

Given the intrinsic capability of NCP‐PEG nanoparticles for noninvasive MR imaging (Figure 3), we explored the feasibility to monitor plaque size change in response to the treatment. In agreement with the histological data (Figure 4C–F), our in vivo MRI showed that, ST/NCP‐PEG treatment could stop atherosclerosis progression (compared with NCP‐PEG) and reduce vessel wall thickness by 14.5% at the end of treatment (Figure 4G,H; Figure S3, Supporting Information), indicating that ST/NCP‐PEG enables real time monitoring of therapeutic efficacy in atherosclerosis treatment in a noninvasive manner. Taken together, the plaque targeting and pH‐responsive properties of ST/NCP‐PEG (Figures 3B,E and 1F) can reduce premature leakage of ST and increase local drug concentration within atherosclerotic plaques, and thereby improving therapeutic efficacy of ST. The conjugated Gd^3+^ allows spontaneous monitoring of the treatment efficacy of ST/NCP‐PEG by noninvasive MRI in vivo.

### 2.5 ST/NCP‐PEG Nanoparticles Enhance Anti‐Inflammatory Activity of Simvastatin In Vivo

**Figure 5:**
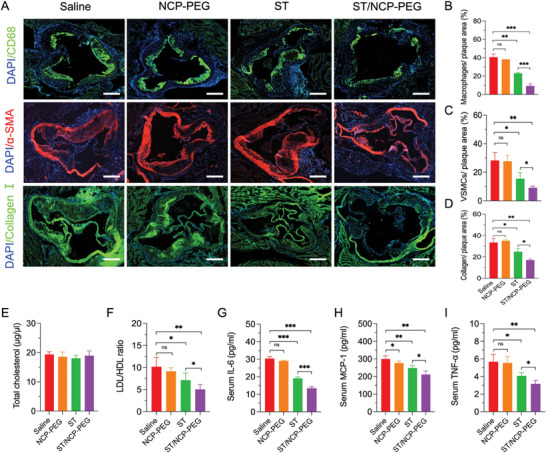
Immunohistochemistry analysis of aortic root sections from ApoE^−/−^ mice receiving different treatments. A) Representative images of aortic root sections stained with antibody to CD68, antibody to α‐SMA, antibody to Collagen I. Quantification of plaque macrophage area relative to total plaque area B), plaque vascular smooth muscle cell (VSMCs) area relative to total plaque area C) and plaque collagen area relative to total plaque area D). Plasma levels of total cholesterol E), LDL/HDL ratio F), IL‐6 G), MCP‐1H) and TNF‐α I). Scale bar = 300 µm. Data is presented as mean ± SD (A–D, *n* = 5; E‐I, *n* = 7; *n* represents the number of mice per group). ns, not significant; *p < 0.05, **p < 0.01, ***p < 0.001.

We further examined composition of the plaques by histochemistry analysis of aortic root cryosections. As indicated by hematoxylin/eosin staining and ORO staining, the acellular necrotic cores in the plaques were filled with lipids (Figure 4E). Compared with NCP‐PEG group or free ST treatment group, mice treated with ST/NCP‐PEG exhibited smaller total area of necrotic cores. Since necrotic cores are formed by apoptotic macrophages while atherosclerotic plaque is advancing,^[^ ^57^ ^]^ reduction in necrotic cores indicates attenuation of atherosclerosis progression by ST/NCP‐PEG. In consistence, we observed marked fewer macrophages (stained by CD68) within the plaques in mouse aortic roots after treatment with either free ST or ST/NCP‐PEG (**Figure** **5**A,B). These results are in agreement with a previous study reporting that ST alleviates atherosclerosis by inhibiting proliferation and infiltration of macrophages.^[^ ^58^ ^]^ Further analysis revealed that macrophage counts were even fewer in the aortic root plaques of ST/NCP‐PEG treatment group than that of free ST treatment group, indicating that delivery by NCP‐PEG nanoparticles improves therapeutic efficacy of ST. As macrophages actively participate in all stages of atherogenesis including monocyte recruitment, foam cell formation, and fibrous cap erosion, reduction in macrophages represents a less inflammatory plaque microenvironment. Since ST has been reported to attenuate atherosclerosis by suppressing both inflammation and oxidative stress, independent of cholesterol‐lowering,^[^ ^6^, ^40^ ^]^ which was confirmed in vitro in this study (Figure 2), we also analyzed ROS contents in the plaques by dihydroethidium assay as previously described.^[^ ^59^ ^]^ As shown in Figure S4 (Supporting Information), mice treated with ST/NCP‐PEG nanoparticles exhibited lower amount of ROS content in the plaques, however, the reduction did not reach statistical significance likely due to the volatile nature of ROS (large amount of ROS may be lost during tissue harvesting and preparation for measuring). Considering the antioxidant activities of ST/NCP‐PEG nanoparticles observed in vitro, our results suggest that ST/NCP‐PEG nanoparticles may attenuate atherosclerosis by diminishing plaque inflammation and oxidative stress.

Vascular smooth muscle cells (VSMCs) stay in artery tunica media under healthy status, whereas pro‐inflammatory cytokines from inflammatory cells such as macrophages within the plaques could induce VSMCs migration to intima where they proliferate and deposit collagen thereby contributing to atherosclerosis pathophysiology during the early stage.^[^ ^57^, ^60^ ^]^ While VSMCs are often considered as an indicator for plaque stability, recent studies indicate that plaque VSMCs may be detrimental in atherosclerosis.^[^ ^60^ ^]^ In particular, plaque VSMCs has been reported to display macrophage‐like phenotype and contribute to inflammation in atherosclerosis.^[^ ^61^ ^]^ In this study, ST treatment decreased accumulation of VSMCs in the intima and collagen deposition in the plaques (Figure 5A,C,D), as indicated by fluorescence staining of α smooth muscle actin (αSMA) and Collagen I, respectively. Moreover, both intima VSMCs and collagen deposition were further reduced by ST/NCP‐PEG nanoparticles. Thes results are seemingly puzzling as some anti‐atherosclerosis therapies increase plaque VSMCs and collagen content.^[^ ^62^, ^63^, ^64^ ^]^ However, the role of VSMCs in atherosclerosis has become controversial^[^ ^60^ ^]^ and reduction in VSMCs/collagen content in early stage of plaque development may be beneficial, contributing to smaller plaque size and less inflammation.^[^ ^65^ ^]^ In fact, ST has been reported to inhibit proliferation and migration of VSMCs,^[^ ^65^, ^66^, ^67^ ^]^ and reduction in VSMCs/collagen content decreases arterial stiffness and thus benefits cardiovascular health.^[^ ^68^ ^]^ Given that VSMCs behave like inflammatory macrophages at certain stages of plaque development, our findings may be ST‐specific, reflecting its anti‐inflammatory and VSMCs inhibitory effects. If this holds true for other stains or anti‐atherosclerotic drugs needs further study.

To examine if plaque‐delivery of ST still has systemic effects, we performed biochemical assays of animal plasma. Consistent with previously reported,^[^ ^6^ ^]^ ST, administered either in free form or in ST/NCP‐PEG nanoparticles, did not influence total cholesterol levels (Figure 5E). Interestingly, ST treatment lowered plasma LDL/HDL ratio, which was further lowered by ST/NCP‐PEG nanoparticle treatment (Figure 5F; Figure S5, Supporting Information). This finding may be explained by the fact that ST does not affect LDL level^[^ ^6^ ^]^ but increases HDL level by upregulating ABCA1 and ApoA‐I which play important roles in HDL maturation in ApoE^−/−^ mice.^[^ ^69^ ^]^ In addition, plasma levels of pro‐inflammatory cytokines (IL‐6, TNF‐α, and MCP‐1) were significantly reduced by free ST and ST/NCP‐PEG nanoparticle treatment with the latter being more effective (Figure 5G–I). These results are consistent with intraplaque reduction of inflammatory macrophages by ST and ST/NCP‐PEG nanoparticles although intraplaque cytokine profile was not determined in the current study (Figurea 5A,B and **6**). Nonetheless, the plasma profile of cytokines further confirms previous notion that ST exerts anti‐atherosclerosis effects via its anti‐inflammatory and antioxidative activities independent of cholesterol lowering.^[^ ^4^, ^6^ ^]^ To understand if the systemic effects of ST/NCP‐PEG nanoparticles were attributed to their potential uptake by circulating immune cells, we performed FACS analysis on peripheral blood cells at 2 h post intravenous administration of Cy5‐labeled NCP‐PEG nanoparticles. As shown in Figure S6 (Supporting Information). Cy5‐labeled NCP‐PEG nanoparticles were not taken up by blood phagocytes, including lymphocytes, neutrophils, as well as Ly‐6C^low^ and Ly‐6C^high^ monocytes. This is probably not unexpected as the hydrophilic and flexible nature of PEG prevents nanoparticles from interacting with the opsonin proteins, and thereby helping PEG‐grafted nanoparticles escape from phagocytic clearance in blood circulation. As a result, ST/NCP‐PEG nanoparticles increased half‐life of ST in the blood stream (Figure S7, Supporting Information) and enhanced its accumulation in the plaque (Figure 3E). Taken together, those data demonstrate that specific inhibition of plaque lesional macrophages by nanoparticle‐delivered ST enhances anti‐atherosclerotic efficacy by reducing both local and systemic atherogenesis risk factors.

**Figure 6:**
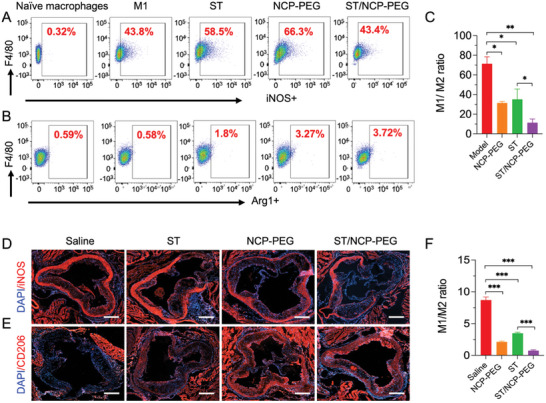
Modulation of the macrophage phenotype by ST/NCP‐PEG nanoparticle treatment. Representative flow cytometric dot plots illustrating the number of A) M1 (F4/80+iNOS+) and B) M2 (F4/80+Arg1+) macrophages after different in vitro treatments. C) M1/M2 ratio of macrophages receiving different in vitro treatments. Immunofluorescence images showing expressions of iNOS D) and CD206 E) in aortic root sections. F) Quantitative analysis of the relative M1/M2 ratio in plaques. Scale bar = 300 µm. Data is presented as as mean ± SD (A‐C, *n* = 3, *n* represents the number of repetitions per group; D‐F, *n* = 5, *n* represents the number of mice per group). ns, not significant; *p < 0.05, **p < 0.01, ***p < 0.001.

### 2.6 ST/NCP‐PEG Nanoparticles Promote Macrophage Polarization toward an Anti‐Inflammatory Phenotype

Apart from reduction in number of macrophages in the plaque, we hypothesize that ST/NCP‐PEG nanoparticles may promote macrophage polarization toward an anti‐inflammatory phenotype since ST/NCP‐PEG nanoparticles inhibited expression of MCP‐1 both in vitro and in vivo (Figures 2G and 5H). In fact, deficiency of MCP‐1 promotes macrophages polarizate toward an anti‐inflammatory phenotype and become M2 macrophages, and thereby alleviating atherosclerosis progression.^[^ ^70^ ^]^ As M1 macrophages primarily secrete proinflammatory cytokines and ROS, whereas M2 macrophages produce anti‐inflammatory cytokines and contribute to tissue repair, enrichment of M2 macrophages makes plaques less inflammatory and more stable. Since macrophage polarization is a highly dynamic and reversible process, the M1/M2 ratio is often chosen to indicate the overall polarization status of macrophages. To address our hypothesis, we first determined M1/M2 ratios of RAW264.7 macrophages following various treatments. As shown in **Figure** **6**C, the M1 cell model group was generated by incubation with LPS and IFN‐γ for 24 h, resulting in polarization of many macrophages into M1 phenotype (F4/80^+^iNOS^+^ cells) and a high M1/M2 ratio. Upon treatment of those M1 model cells with ST or NCP‐PEG nanoparticles, the M1/M2 ratio was decreased by ≈50%. Treatment with ST/NCP‐PEG nanoparticles further reduced the M1/M2 ratio, by 85% of the model group (Figure 6C). Similarly, as revealed by immunohistochemistry analysis, ApoE^−/−^ mice receiving ST/NCP‐PEG nanoparticles treatment exhibited markedly lower M1/M2 ratio in atherosclerotic plaques of aortic roots than those receiving NCP‐PEG nanoparticles or ST treatment (Figure 6D–F). These results confirm that ST is able to shift macrophage phenotype from M1 to M2. In agreement with our findings, it has been reported that ST inhibits expression of pro‐inflammatory cytokines^[^ ^71^ ^]^ by regulating the Notch signaling pathway.^[^ ^72^, ^73^ ^]^ Moreover, since NADPH oxidase is activated in M1 macrophages to produce ROS, polarization of macrophages toward M2 phenotype may explain the reduction in ROS by ST and ST/NCP‐PEG nanoparticles in RAW264.7 macrophages observed in our in vitro study. The reduction in M1/M2 ratio by NCP‐PEG nanoparticles compared to saline control is a bit surprising but likely due to the immuno‐modulatory property of PEG.^[^ ^74^, ^75^ ^]^ As a result, the synergistic effects of ST and NCP‐PEG nanoparticles empower ST/NCP‐PEG nanoparticles more potent activity to polarize macrophages toward M2 phenotype than free ST (Figure 6C,F). However, NCP‐PEG nanoparticles alone were not sufficient to reduce plaque size in vivo (Figure 4C–F). This discrepancy may be because NCP‐PEG nanoparticles are unable to inhibit expression of a broad range of proinflammatory cytokines except MCP‐1 (Figure 2) or suppress recruitment of macrophages to the plaque (Figure 5A,B). As a result, the generated M2 macrophages in vivo by NCP‐PEG nanoparticles alone are not enough to attenuate plaque development (Figure 4C–F). Thus, the anti‐atherosclerosis effects of ST and ST/NCP‐PEG nanoparticles are mainly attributable to reduction in M1 macrophage counts but amplified by macrophage polarization.

### 2.7 Safety Assessment of ST/NCP‐PEG Nanoparticles in ApoE^−/−^ Mice

Finally, we investigated the potential toxicity of ST/NCP‐PEG nanoparticles. Animal body weight increased in all groups over the 8‐week treatment period and no obvious difference was observed between saline and treatment groups (Figure S8A, Supporting Information). The blood biochemical assays did not exhibit significant increase in aspartate aminotransferase (AST), alanine aminotransferase (ALT), serum creatinine (Scr), or blood urea nitrogen (BUN), indicating long‐term treatment with ST/NCP‐PEG nanoparticles did not affect hepatic and renal function (Figure S8B, Supporting Information). Moreover, as demonstrated by histological analysis of cryosections, no obvious pathological changes were observed in major organs (Figure S8C, Supporting Information). Taken together, these results demonstrate that long‐term intravenous administration of ST/NCP‐PEG nanoparticles was safe at the therapeutic dosage.

## 3 Conclusion

In this study, we have synthesized novel NCPs composed of pH‐responsive linker and Gd^3+^ and developed a biocompatible, biodegradable, biologically safe, and plaque microenvironment‐responsive NCP‐PEG nanoparticle‐based theranostic nanomedicine for spontaneous plaque imaging and atherosclerosis treatment. ST was successfully loaded into the hydrophobic domain of lipid bilayer shell on the surface of NCPs to obtain ST/NCP‐PEG nanoparticles. Upon intravenous administration, ST/NCP‐PEG nanoparticles could efficiently accumulate within atherosclerotic plaques and disintegrate in response to the plaque mild acidic conditions. The released Gd‐complex provided heightened *T_1_*‐weighted MRI signal for diagnosis purpose. Simultaneously, ST exerted localized anti‐inflammatory and antioxidative effects. In vivo therapeutic study indicates that ST/NCP‐PEG nanoparticles could attenuate atherosclerosis progression more efficiently than free ST, as ST/NCP‐PEG nanoparticles allows for specific delivery and longer circulation time of ST. Preliminary safety assessment suggests that ST/NCP‐PEG nanoparticles is safe for long‐term use. To conclude, ST/NCP‐PEG nanoparticles represents a significant advancement in developing theranostic nanomedicine for simultaneous diagnosis and treatment of atherosclerosis, which may inspire nanomedicine development for other disease conditions.

## 4 Experimental Section

### Materials

GdCl_3_·6H_2_O, Glycine, KOH, triethylamine (TEA), TEREPHTHALALDEHYDE (TPH), Chloroform, TritonTM X‐100, Cyclohexane, IGEPAL CO‐520, 1‐Hexanol, simvastatin, and lipopolysaccharides (LPS) were purchased from Sigma‐Aldrich (USA). Cholesterol, 1, 2‐dioleoyl‐sn‐glycero‐3‐phosphate (DOPA), 1, 2‐dihexadecanoyl‐sn‐glycero‐3‐phosphocholine (DPPC), and 1,2‐distearoyl‐sn‐ glycero‐3‐phosphoethanol‐amine‐N‐(methoxy(polyethylene glycol)−5000) (DSPE‐mPEG5k) were purchased from Avanti Polar Lipids (USA). Cyanine5 NHS ester (Cy5) was purchased from Lumiprobe (USA). Penicillin, streptomycin, fetal bovine serum (FBS), and Dulbecco's modified Eagle's medium (DMEM) were purchased from Gibco (USA). F4/80, iNOS, Arg1, Ly6C, Ly6G, CD3, and CD11c antibodies as well as Aspartate Aminotransferase (AST) and Alanine Transaminase (ALT) activity assay kit were purchased from Abcam (the UK). IL‐6, TNF‐α and MCP‐1 ELISA kits were purchased from Biolegend (USA). CD68 and CD206 primary antibodies were purchased from Bio‐Rad (USA). Collagen Type 1 was purchased from Merck Millipore (USA). Total cholesterol fluorometric assay kit was purchased from Biovision (USA). Di(Acetoxymethyl Ester) (6‐Carboxy‐2′,7′‐Dichlorodihydrofluorescein Diacetate) (DCFH‐DA) kit, IFN‐γ, Serum creatinine (Scr), blood urea nitrogen (BUN) kits were purchased from Thermo Fisher (USA).

### Synthesis of NCP‐PEG and ST/NCP‐PEG Nanoparticles

pH‐responsive benzoic‐imine (BI) linker was first synthesized as part of the Nanoscale coordination polymer (NCP) component. Briefly, 4 mmol glycine and 4 mmol KOH were dissolved in 70 ml absolute ethanol overnight. Terephthalaldehyde (TPH) (2 mmol) was gradually added into the above suspension and reflux at 120 °C for 2 h. The final product was washed with absolute ethanol three times under centrifugation at 15 000 rpm. NCPs were synthesized through reverse microemulsion method as previously described^[^ ^22^, ^25^ ^]^ with adaptions. Two microemulsions were prepared by separately adding 75 µl GdCl_3_ aqueous solution or 75 µl BI‐linker aqueous solution into the 2.5 ml microemulsion reaction buffer composing 0.65 m TritonTM X‐100/1.23 m 1‐Hexanol/3.395 ml Cyclohexane /0.0732 m IGEPAL CO‐520 under vigorous stirring at room temperature. The two microemulsions were then mixed with 5 µl triethylamine (TEA) and stirred for another 10 min at 650 rpm. The resultant 5 ml microemulsions were subsequently added with 5 ml absolute ethanol. NCP nanoparticles were obtained by centrifugation at 15 000 rpm for 5 min. The obtained NCP nanoparticles were washed twice with absolute ethanol and were redispersed into absolute ethanol for further use. The NCP‐PEG nanoparticles synthesis procedure could be divided into two steps. First, 2 mg (0.2 mg ml^−1^) NCP nanoparticles in ethanol was mixed with 0.2 mg (0.2 mg ml^−1^) DOPA in chloroform for 30 min under intense ultrasonication. Then the solution was centrifuged, and the precipitant was washed twice with ethanol to remove the unconjugated DOPA and redispersed in chloroform. PEGylation of NCP was performed by sequentially adding chloroform solution containing DPPC, cholesterol and DSPE‐mPEG_5k_ with a mole ratio of 2:2:1 into NCP‐DOPA chloroform solution under intense ultrasonication and stirred overnight. Afterward, chloroform was evaporated and the obtained nanoparticles were denoted as NCP‐PEG nanoparticles and stored at 4 °C for further use.

For ST loading, various mole ratios of DSPE‐mPEG_5k_ and ST (1:0.5, 1:1, 1:1.5, 1:2) were added into the reaction system as in the above second step. The excessed ST was removed by washing with 100 kDa ultracentrifugal filter three times and the constructed ST/NCP‐PEG complex was stored at 4 °C in PBS for further use. Drug loading capacity was calculated according to the following formula.

(1) $$\mathrm{DL}\%\;=\;m_{2}\;/\;\left(m_{1}+m_{0}\right)\;x\;100\%$$

m_0_, m_1_, and m_2_ refers to the total mass of NCP‐PEG nanoparticles added to the reaction system, the total mass of drug put in the reaction system and the loaded mass of ST, respectively.

Additionally, to prepare the Cy5 labeled NCP‐PEG nanoparticles, 2 µl of Cy5 NHS ester (1 mg ml^−1^) DMSO solution was added together with chloroform solution containing DPPC, cholesterol, and DSPE‐mPEG5k into NCP‐DOPA chloroform solution to construct Cy5/NCP‐PEG nanoparticles. The mole ratio of DPPC, cholesterol, Cy5 NHS ester, and DSPE‐mPEG5k was 2:2:0.34:1.

### Characterization of the ST/NCP‐PEG Nanoparticles

The morphology and size of NCP‐PEG nanoparticles were characterized by TEM (JEOL 2200FS Cryo TEM, JEOL USA, Inc.) and DLS (Nanosizer, Malvern Instruments Ltd., Worcestershire, UK), respectively. Nanoparticle composition and content of Gd element were investigated by TGA and ICP‐MS. The concentration‐dependent MRI phantom study was validated with a 7.0 T MRI system (SIEMENS, Germany). A mouse macrophage cell line (RAW264.7) were seeded in a black 96‐well plate at a density of 10^4^ cells well^−1^ in 100 µl comprehensive (cDMEM) to evaluate the cytotoxicity of NCP‐PEG nanoparticles. Cells were treated with various concentrations of NCP‐PEG nanoparticles for different time interval and cell viability was measured with PrestoBlue.

In order to evaluate pH‐responsive drug release performance, 0.5 mg ml^−1^ ST/NCP‐PEG nanoparticles were evenly dispersed into 10 ml pH 5.6 or pH 7.4 PBS at 37 °C. An equal volume of release medium was removed at indicated time intervals and measured by HPLC at a constant wavelength of 238 nm. Similarly, released Gd ions within the supernatant was determined by ICP‐MS.

### Cellular Uptake of Cy5/NCP‐PEG

0.5 × 10^6^ cells well^−1^ were seeded into the 12‐well plate overnight. Activated cells were treated with LPS/IFNγ (300 and 20 ng ml^−1^, respectively) for 24 h and nonactivated cells were treated with fresh cDMEM for 24 h. Cells from the two groups were treated with 25 µg ml^−1^ Cy5/NCP‐PEG nanoparticles for different time periods (2‐8 h). Thereafter, the cells were trypsinized for FACS (BD Bioscience, USA) analysis.

For microscopic analysis, RAW264.7 cells were seeded into the 12 well plate with a density of 0.1 × 10^6^ cells well^−1^ overnight. The cells were then treated the same as above. After different incubation period, the cells were rinsed 3 times and fixed with 4% paraformaldehyde for 10 min, and DAPI was applied to stain the nuclei. The fixed cells were then observed with an inverted fluorescence microscope (ECLIPSE *Ti*, NIKON, Japan).

### Cellular Uptake of NCP‐PEG Nanoparticles in Vivo

ApoE^−/−^ mice fed on HFD for more than 8 weeks were injected intravenously with Cy5‐labeled NCP‐PEG nanoparticles. 24 h post‐injection, aortic roots were harvested for cryosections. Immunofluorescence staining with CD68 (macrophage marker) was performed on the aortic root sections to visualize the uptake of nanoparticles in the plaque macrophages.

### In Vitro Anti‐Inflammatory Effects in Macrophages

RAW264.7 cells were seeded into the 96 well plate at a concentration of 10^5^ cells/100 µl well^−1^ overnight. The cells in the control group were replaced with fresh cDMEM and the cells in the model group were stimulated with 300 ng ml^−1^ LPS + 20 ng ml^−1^ IFN‐γ for 24 h. The rest of the groups were co‐treated with 300 ng ml^−1^ LPS + 20 ng ml^−1^ IFN‐γ + ST, 300 ng ml^−1^ LPS + 20 ng ml^−1^ IFN‐γ + ST/NCP‐PEG nanoparticles, and 300 ng ml^−1^ LPS + 20 ng ml^−1^ IFN‐γ + NCP‐PEG nanoparticles in cDMEM for 24 h, at an identical ST dose of 24 µm. Subsequently, the supernatants were collected by centrifugation at 1000 rpm for 5 min. Typical inflammatory cytokines, including IL‐6, IL‐1β, and TNF‐α were determined by ELISA kit according to the protocol.

### Antioxidant Effects in Macrophages

RAW264.7 cells were seeded into the 12‐well plate at 0.5 × 10^6^ cells well^−1^ and incubated overnight. The control group was treated with fresh cDMEM and the model group was stimulated by 300 ng ml^−1^ LPS + 20 ng ml^−1^ IFN‐γ for a 4 h. The rest the groups were co‐treated with LPS/IFN‐γ + ST, LPS/IFN‐γ + ST/NCP‐PEG nanoparticles, and LPS/IFN‐γ + NCP‐PEG nanoparticles in cDMEM for 4 h, at an identical ST dose of 24 µm. Subsequently, cells were stained with DCFH‐DA kit (10 µm) in staining buffer for 30 min at 37 °C. After being washed with staining buffer, intracellular fluorescence was detected via FACS and analyzed by FlowJo software. For microscopic analysis, cells were treated the same as FACS but were stained with DCFH‐DA kit (10 µm) for 45 min in cell‐culture plate and observed by an inverted fluorescent microscope.

For ROS detection in plaque tissue, briefly, serial aortic root sections were cut at 8 µm thick using a cryostat at −20 °C. The slides were placed in the 5 µm DHE staining solution and incubated for 5 min at room temperature in the dark. The slides were rinsed twice in Milli‐Q water (1 min each) and mounted with DAPI mounting media. The slides were imaged immediately with an inverted fluorescence microscope (ECLIPSE Ti, NIKON, Japan). DHE‐derived 2‐OH‐E+ could be visualized with a 594 nm excitation filter, and the autofluorescence of elastin in the internal elastic lamina was captured in the GFP channel.

### Modulation of Macrophage Polarization by ST/NCP‐PEG Nanoparticle Therapy

The effect of ST/NCP‐PEG nanoparticles on in vitro macrophage polarization was investigated. RAW264.7 cells were first polarized into M1 phenotype by treating with 300 ng ml^−1^ LPS + 20 ng ml^−1^ IFN‐γ for 24 h, and then were treated with ST, ST/NCP‐PEG or NCP‐PEG formulations at an identical ST dosage of 24 µm for another 24 h. Subsequently, the cells were harvested and stained with pacBlue, anti‐F4/80, anti‐iNOS and anti‐Arg1 antibodies. After being washed with PBS, cells were analyzed by FACS.

### Animals

Animal care and experiments were carried out in accordance with the National Advisory Committee for Laboratory Animal Research (NACLAR) Guidelines. All protocols and procedures had been approved by National University of Singapore Institutional Animal Care and Use Committee (IACUC protocol: 2021‐01032). Basically, ApoE^−/‐^ mice were housed under specific pathogen‐free (SPF) conditions and given a 12 h light/dark cycle with ad libitum access to food and water.

### In Vivo Biodistribution of Nanoparticles

Four‐week‐old male ApoE^−/−^ mice were fed with a high‐fat diet (HFD) containing 42% kcal from fat for 12 consecutive weeks. Then Cy5/NCP‐PEG nanoparticles were administrated by i.v. injection at 4.5 mg kg^−1^. At 3, 6, and 24 h after administration, mice were perfused with 10 ml PBS under anesthesia by 1.5% isoflurane. Subsequently, aorta and major organs (heart, liver, spleen, lung, kidney) were harvested. IVIS (in vivo imaging system) spectrum system (PerkinElmer, USA) was carried out to perform the ex vivo imaging and mean fluorescence intensity (MFI) was analyzed by the Living Image 4.5 software.

### In Vivo 7 Tesla MRI

For in vivo MRI imaging, abdominal aorta region of ApoE^−/−^ mice on 12 weeks HFD were scanned with a 7.0 T MRI system (SIEMENS, Germany) before and after i.v. administration of NCP‐PEG nanoparticles at the concentration of 4.5 mg kg^−1^. Five consecutive 1000‐µm‐thick slices were captured using a spin echo sequence. A microscale in‐plane resolution of 192 µm was achieved. The TR (repetition time) and TE (echo time) were 874.94 and 1.05 ms, respectively. The flip angle was 70° and the field of view was 40 × 40 mm. Outer wall area (OWA) and aortic lumen area were determined with NIS‐Element AR Analysis (NIKON, Japan). The mean wall area (MWA) was defined as the difference between lumen area and OWA. Normalized wall index (NWI) was the primary outcome parameter, which was calculated as: NWI = MWA/OWA.

### LC‐MS Measurement of ST

For generation of the standard curve for detecting ST, ST was weighted and dissolved in acetonitrile solution; a series of ST standard solutions at different concentrations were obtained by stepwise dilutions. The signal intensity of the ST solutions was detected by HPLC‐MS, thereby establishing a ST concentration standard curve. ST with different concentrations was dissolved in blank plasma, then the drug was extracted into acetonitrile using the above method and detected by HPLC‐MS. The ratio of the obtained curve to the ST standard curve was the recovery rate, which was ≈78%.

Pharmacokinetics of Simvastatin. 12 eight‐week‐old C57BL/J mice were grouped into two groups to receive either ST (dissolved in DMSO and further diluted with saline) or ST/NCP‐PEG nanoparticles at an equal drug concentration of 2 mg kg^−1^ ST. Blood was collected via submandibular cheek puncture at 30‐, 60‐, 120‐, 240, and 1440 min post‐injection and plasma was prepared for LC‐MS measurement of ST. Ten microliters of plasma was dissolved in 100 µL of acetonitrile solution, filtered using a 0.22 µm filter after sufficient ultrasonication in water bath. The solution was used for HPLC‐MS measurement.

Detection of simvastatin content in atherosclerotic plaques. 6 ApoE mice fed with HFD for more than 8 weeks were injected intravenously with either free ST or ST/NCP‐PEG nanoparticles at an equal drug concentration of 2 mg kg^−1^ ST. Twenty‐four hours post‐injection, the mice were euthanized, and aorta tissue was isolated for LC‐MS measurement of ST. The aorta with plaques was weighed and crushed in liquid nitrogen using a manual homogenizer. Two hundred microliters of acetonitrile solution was added and mixed with tissue lysate. ST was fully extracted into the organic phase by ultrasound. The solution was filtered through a 0.22 µm filter for subsequent HPLC‐MS measurement.

### Treatment of Atherosclerosis by ST/NCP‐PEG Nanoparticles in ApoE^−/−^ Mice

Four‐week‐old male ApoE^−/−^ mice were fed with the HFD for 12 consecutive weeks. They were randomly divided into 4 groups (*n* = 7) after the first 4 week of HFD, followed by different treatments for an additional 8 weeks. Mice in the control group were treated with saline, while the rest groups received NCP‐PEG nanoparticles at 4.5 mg kg^−1^, free ST, or ST/NCP‐PEG nanoparticles at an identical ST dose of 2 mg kg^−1^. Both NCP‐PEG and ST/NCP‐PEG nanoparticles were dispersed into saline, ST was first dissolved in DMSO followed by dilution in saline (DMSO concentration in the final solution is lower than 0.1%). All formulations were given once weekly.

### Quantification of Therapeutic Outcome in ApoE^−/−^ Mice

Atherosclerotic plaque size in the whole aorta and aortic root was determined by ORO staining as previously described.^[^ ^54^ ^]^ In brief, the whole aorta starting from the left common carotid artery to the iliac bifurcation was collected and fixed in 10% formalin for a minimum of 72 h before proceeding with ORO staining. Additionally, serial sections of aortic roots were obtained and stained with ORO. Plaque area in aortas and aortic roots were individually analyzed and quantitated by Image J (version 1.54).

For immunohistochemistry analysis, cryosections of aortic roots were stained with antibodies to CD68, CD206, CD206, and inducible nitric oxide synthase (iNOS) antibodies. Images were captured with inverted fluorescent microscope and analyzed with NIS‐Element AR Analysis (NIKON, Japan). Only cells located in the region of interest which were demarcated by the plaque area were counted as a percentage of positive cells to the total plaque area.

Whole blood was collected through heart puncture in ApoE^−/‐^ mice after receiving different treatments followed by centrifugation at 4000 g for 10 min to collect the plasma. Proinflammatory cytokines including IL‐6, MCP‐1, and TNF‐α were detected using the commercial ELISA kits. Total cholesterol, low density lipoprotein (LDL), and high density lipoprotein (HDL) were detected with total cholesterol fluorometric assay kit.

### Safety Evaluation

The levels of alanine aminotransferase (ALT), aspartate aminotransferase (AST), serum creatinine (Scr), and blood urea nitrogen (BUN) in blood were measured to evaluate liver and kidney function after long‐term treatment.

### Statistical Analysis

Statistical analysis was performed with GraphPad Prism. Data were presented as mean ± standard deviation (SD) and were analyzed by unpaired two‐tailed t‐test for the comparison of two groups or one‐way analysis of variance (ANOVA) with Bonferroni's multiple comparisons for comparison of three groups or more. Statistical significance was annotated with *P ≤ 0.05, **P ≤ 0.01, and ***P ≤ 0.001.

### Ethical Statement

Animal care and experiments were carried out in accordance with the National Advisory Committee for Laboratory Animal Research (NACLAR) Guidelines. All protocols and procedures had been approved by National University of Singapore Institutional Animal Care and Use Committee (IACUC protocol: 2021‐01032).

## Conflict of Interest

The authors declare no conflict of interest.

## Author Contributions

Y.L., J.L., and S.Y.C. These authors contributed equally. J.W.W. conceived, designed and supervised this study; Y.L., J.L., and S.Y.C. performed most of the experiments; Y.L. and J.L. designed the nanomaterials; Y.L., J.L., P.P., X.H., and Z.Y. contributed to the MR imaging; S.Y.C., H.J.T., S.Z., X.Q., and C.H. performed animal experiments; X.T., L.Y., L.G., and F.T.T. performed LC‐MS measurement of Simvastatin; X.L., B.L., J.C.Y.K. and J.W.W. supervised and coordinated this collaboration; Y.L. and J.W.W. wrote the manuscript. All authors contributed to data analysis, manuscript review and editing.

## Supporting information

Supporting Information

## Data Availability

The data that support the findings of this study are available in the supplementary material of this article.
