# Supplementary material for: Dual‐Function Nanoscale Coordination Polymer Nanoparticles for Targeted Diagnosis and Therapeutic Delivery in Atherosclerosis
Source: Small. 2024 Aug 26;20(47):2401659. doi: 10.1002/smll.202401659 (PMC11579969; doi:10.1002/smll.202401659)
Supplement: Supplementary file 1 — Supporting Information [file SMLL-20-2401659-s001.docx]

Supporting Information

**Dual-Function Nanoscale Coordination Polymer Nanoparticles for Targeted Diagnosis and Therapeutic Delivery in Atherosclerosis**

Yuanzhe Lin^a,b,c,1^, Jingjing Liu^d,e,1^, Suet Yen Chong^a,c,f,1^, Hui Jun Ting^a,c^, Xichuan Tang^a,c^, Liqiang Yang^a,c^, Sitong Zhang^a,c^, Xinyi Qi^a,c^, Peng Pei^a,c,g^, Zhigao Yi^g^, Chenyuan Huang^a,c^, Xiao Hou^a,c^, Liang Gao^h, i^, Federico Torta^h, i^, Xiaogang Liu^g^, Bin Liu^e,*^, James Chen Yong Kah^b,*^, and Jiong-Wei Wang^a,c,f,j*^

^a^ Department of Surgery, Yong Loo Lin School of Medicine, National University of Singapore, 1E Kent Ridge Rd, Singapore 119228, Singapore

*Email: surwang@nus.edu.sg

^b^ Department of Biomedical Engineering, National University of Singapore, 4 Engineering Drive 3, Block E4, #04-08, Singapore 117583, Singapore

*Email: kah@nus.edu.sg

^c^ Nanomedicine Translational Research Program, Yong Loo Lin School of Medicine, National University of Singapore, 30 Medical Drive, Singapore 117609, Singapore

^d^ Institute of Translational Medicine, Medical College, Yangzhou University, Yangzhou, 225001, Jiangsu, China

^e^ Department of Chemical and Biomolecular Engineering, National University of Singapore, Singapore 117585, Singapore

*Email: cheliub@nus.edu.sg

^f^ Cardiovascular Research Institute, National University Heart Centre Singapore (NUHCS), 14 Medical Drive, Singapore 117599, Singapore

^g^ Department of Chemistry, National University of Singapore, 3 Science Drive 3, Singapore 117543, Singapore

^h^ Department of Biochemistry, Yong Loo Lin School of Medicine, National University of Singapore, 8 Medical Drive, Singapore 117596, Singapore.

^i^ Singapore Lipidomics Incubator (SLING), Life Sciences Institute, National University of Singapore, 28 Medical Drive, Singapore 117456, Singapore

^j^ Department of Physiology, Yong Loo Lin School of Medicine, National University of Singapore, 2 Medical Drive, Singapore 117593, Singapore

^1^ These authors contributed equally.

Corresponding author: Jiong-Wei Wang

Email address: [surwang@nus.edu.sg](mailto:surwang@nus.edu.sg)

Tel.: +65-6601-1387

**Experimental Section**

*LC-MS/MS analysis*

The LC-MS/MS analysis was performed on an Agilent UHPLC 1290 Infinity II liquid chromatography system connected to an Agilent QqQ 6495D.

*LC.* An Agilent Zorbax RRHD Eclipse Plus C18 column (2.1 × 50 mm, 1.8 µm) was used for the RPLC separation. The mobile phases A (water with 0.1% formic acid) and B (acetonitrile with 0.1% formic acid) were used for the chromatographic separation. The following gradient was applied: 0-1 min, 20% B; 1-6 min, 20-100% B; 6-8 min, 100% B; 8.01-10 min, 20% B. The oven temperature was maintained at 40°C. Flow rate was set at 0.6 mL/min and the sample injection volume was 5 µL.

*QqQ.* The positive ionization spray voltage and nozzle voltage were set at 3,000 V and 0 V, respectively. The drying gas and sheath gas temperatures were maintained at 250°C and 400°C, respectively. The drying gas and sheath gas flow rates were 16 L/min and 12 L/min, respectively. The nebulizer nitrogen gas flow rate was set at 40 psi. The iFunnel high and low pressure RF were 150 V and 60 V, respectively. Simvastatin was monitored by MRM transition 419.3 -> 198.9 under positive ionization mode.

**Supplemental Figures**


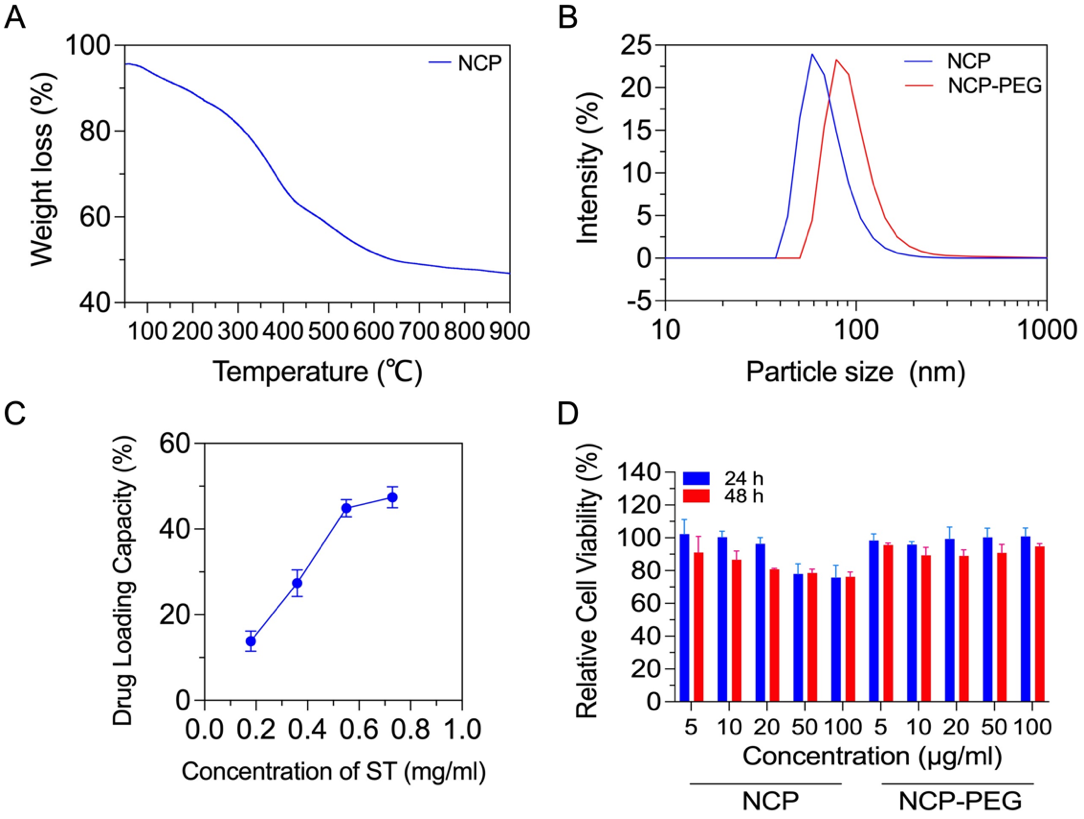


**Figure S1.** Characterization of NCP and NCP-PEG nanoparticles. (A) Thermogravimetric analysis (TGA) curves of nanoscale coordination polymer (NCP) nanoparticles. (B) Hydrodynamic diameter (D_h_) of NCP and NCP-PEG. (C) The ST loading capacity of NCP-PEG at various drug feeding concentrations. (D) Cell viability of RAW264.7 cells incubated with NCP or NCP-PEG nanoparticles for different time periods. Data is presented as mean ±SD (C, n = 3; E, n = 5; n represents the number of repetitions per group).


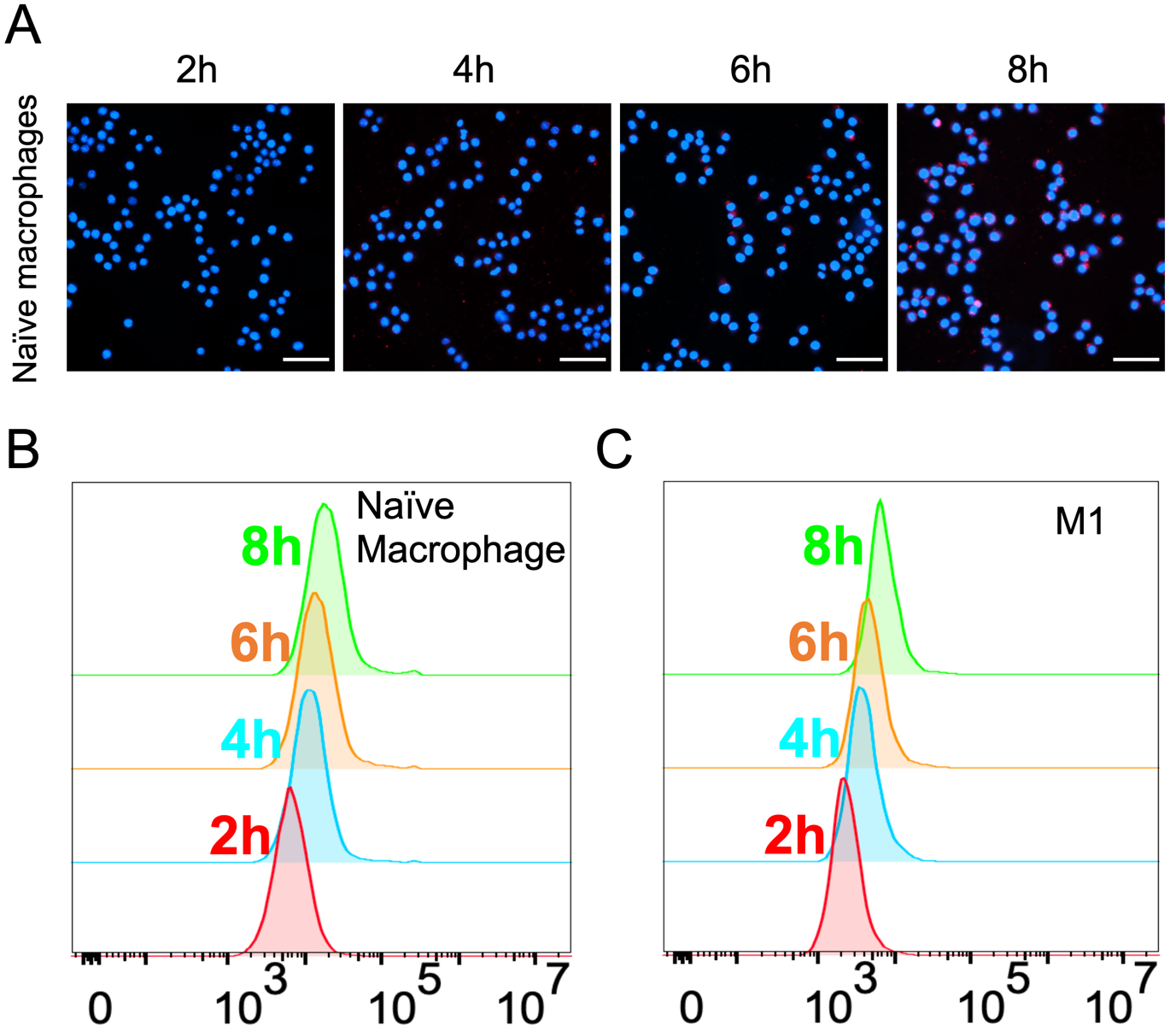


**Figure S2.** Time-dependent uptake profile of Cy5-labelled NCP-PEG nanoparticles by macrophages. (A) Representative images of naïve macrophages treated with Cy5-labeled NCP-PEG nanoparticles at different timepoints. Cell nuclei were stained in blue; Cy-5 NCP-PEG nanoparticles were shown in red. Scale bar = 50 μm. (B,C) Representative illustrations of FACS analysis of cellular uptake by macrophages.


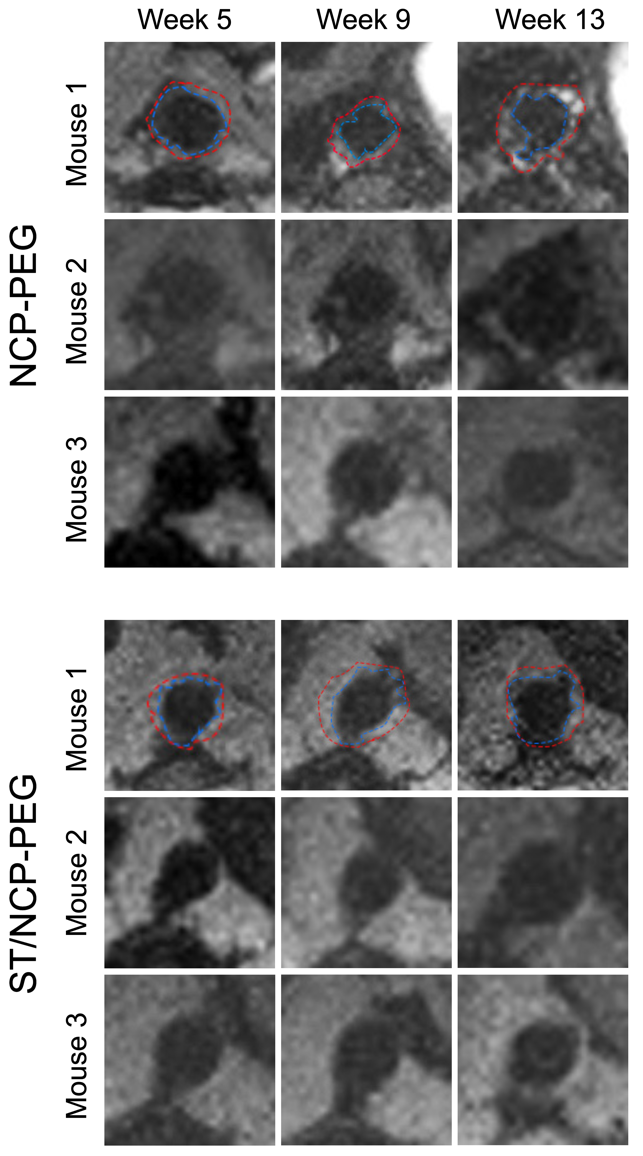


**Figure S3.** Raw *T_1_*-weighted MR images of abdominal aorta of all ApoE^-/-^ mice receiving either NCP-PEG or ST/NCP-PEG at different timepoints. The vessel wall thickness was demarcated by red and blue dotted lines. Supplemental data to Figure 4G.

**
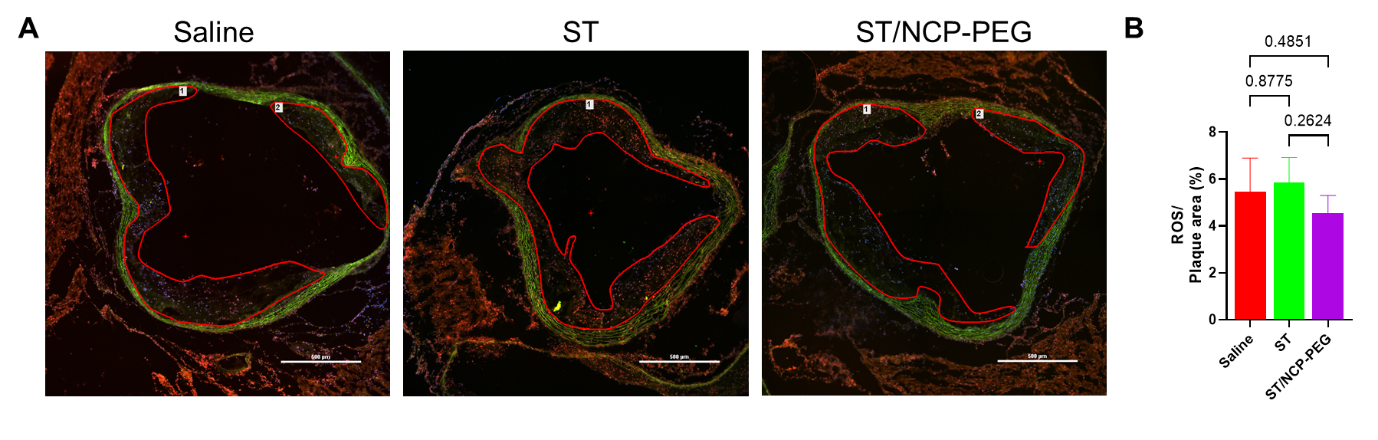
Figure S4.** ROS analysis in aortic tissue. (A) Representative images of aortic root sections stained with Dihydroethidium (DHE) in red for ROS quantification. Auto-fluorescence of the elastin was captured in the green channel as a tissue reference. Cell nuclei were stained with DAPI. Red demarcation represents the aortic plaques. Scale bar = 500 µm. (B) Quantification of ROS in the plaques (n = 4).


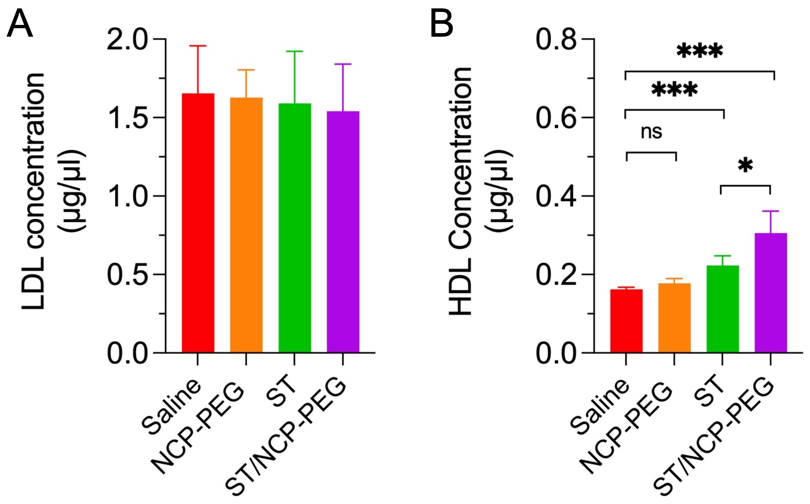


**Figure S5.** Plasma levels of different lipoproteins. (A) low density lipoprotein (LDL), (B) high density lipoprotein (HDL). Data is presented as mean ± SD (A-B, n = 7; n represents the number of mice per group). ns, not significant; **p*<0.05, ****p*<0.001.


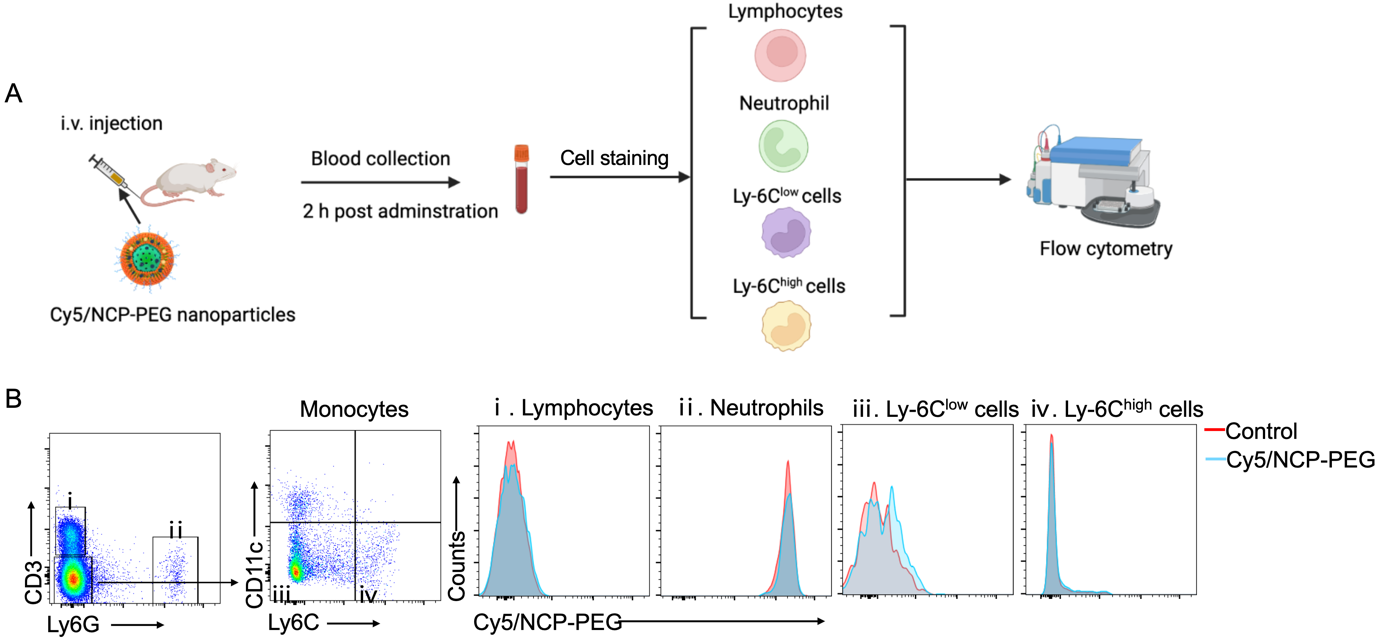


**Figure S6.** Distribution of Cy5/NCP-PEG nanoparticles in blood cells upon i.v. injection. (A) Schematic illustration of blood cell sorting post i.v. injection of Cy5/NCP-PEG nanoparticles. (B) Distribution of Cy5/NCP-PEG nanoparticles in blood cells after i.v. injection in ApoE^-/-^ mice (n = 5, n represents the number of mice per group).


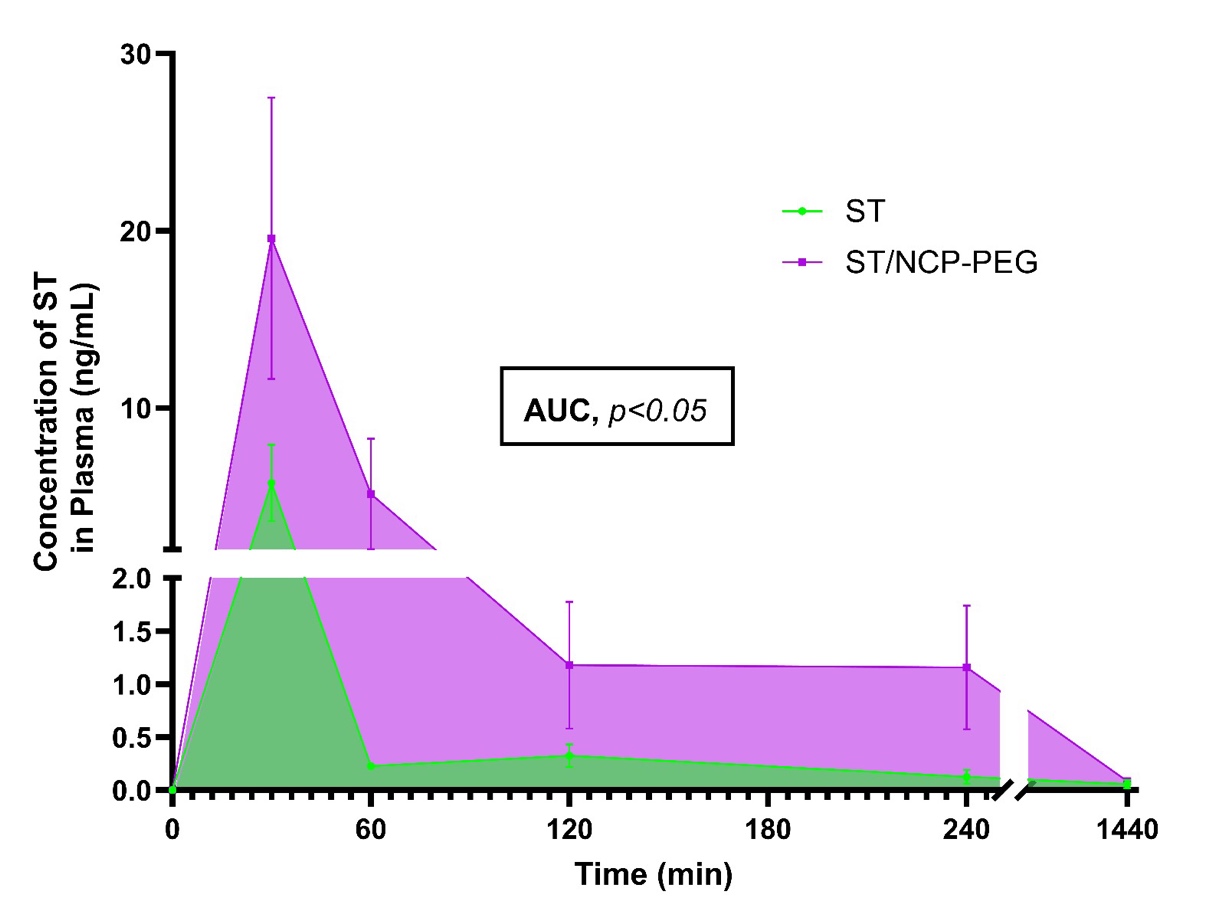


**Figure S7.** Pharmacokinetic profile of Simvastatin. Healthy C57BL/J mice were intravenously administered with either free ST (dissolved in DMSO and diluted with saline) or ST/NCP-PEG nanoparticles at an equal drug concentration of 2mg/kg. Blood was collected via submandibular cheek puncture at designated timepoints (30, 60, 120, 240 and 1440 minutes) post administration. Plasma concentration of ST was determined by LC-MS approach. Data is presented as mean ± SEM (n = 3 mice per group). AUC, area under curve.


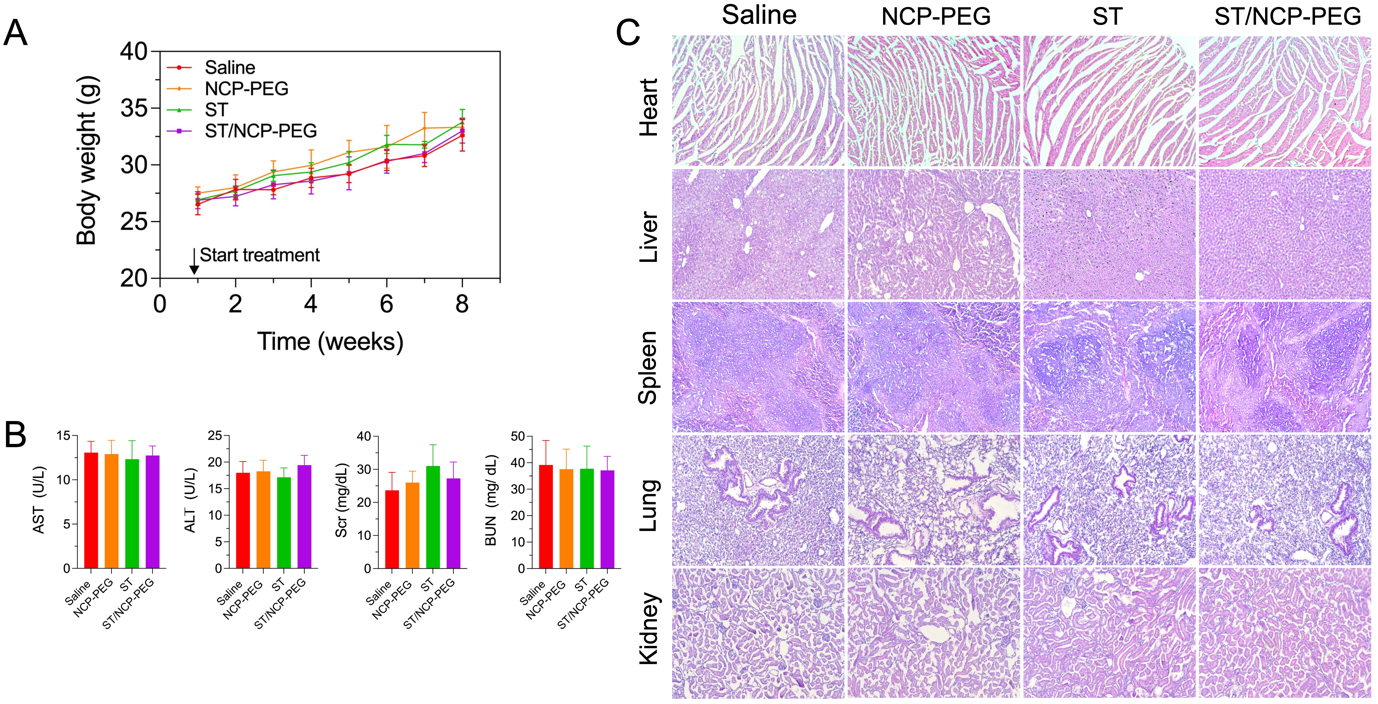


**Figure S8.** Safety evaluation after 8-weeks treatment. (A) The body weight of ApoE−/−mice receiving different formulations. (B) Levels of typical biochemical markers relevant to hepatic (AST and ALT) and kidney (Scr and BUN) functions in plasma. H&E staining of major organ cryosections (C). Data is presented as mean ±SD (A, n = 10; B, n = 7; n represents the number of mice per group).
